# A short isoform of ATG7 fails to lipidate LC3/GABARAP

**DOI:** 10.1038/s41598-018-32694-7

**Published:** 2018-09-26

**Authors:** M. H. Ogmundsdottir, V. Fock, L. Sooman, V. Pogenberg, R. Dilshat, C. Bindesbøll, H. M. Ogmundsdottir, A. Simonsen, M. Wilmanns, E. Steingrimsson

**Affiliations:** 1 Department of Biochemistry and Molecular Biology, Biomedical Center, Faculty of Medicine, University of Iceland, Sturlugata 8, 101 Reykjavik, Iceland; 2 European Molecular Biology Laboratory, Hamburg Unit, Notkestrasse 85, 22607 Hamburg, Germany; 3 Department of Molecular Medicine, Institute of Basic Medical Sciences, University of Oslo, Sognsvannsveien 9, N-0317 Oslo, Norway; 4 Cancer Research Laboratory, Biomedical Center, Faculty of Medicine, University of Iceland, Sturlugata 8, 101 Reykjavik, Iceland

## Abstract

Autophagy is a degradation pathway important for cellular homeostasis. The E1-like enzyme ATG7 is a key component of the autophagy machinery, with the main function of mediating the lipidation of LC3/GABARAP during autophagosome formation. By analysing mRNA-sequencing data we found that in addition to the full-length *ATG7* isoform, various tissues express a shorter isoform lacking an exon of 27 amino acids in the C-terminal part of the protein, termed *ATG7(2)*. We further show that ATG7(2) does not bind LC3B and fails to mediate the lipidation of members of the LC3/GABARAP family. We have thus identified an isoform of ATG7 that is unable to carry out the best characterized function of the protein during the autophagic response. This short isoform will have to be taken into consideration when further studying the role of ATG7.

## Introduction

Autophagy is a highly conserved clearance pathway of cytoplasmic constituents and is essential for sustaining cellular homeostasis^1^. During this catabolic process, cellular components such as protein aggregates, lipids or damaged organelles are delivered to lysosomes, where they become degraded and recycled into building blocks for new cellular constituents and for ATP production^2^. The autophagic response is elicited by various stress conditions, ranging from nutrient deprivation to microbial invasion. Several types of autophagy have been described, namely macroautophagy, microautophagy and chaperone-mediated autophagy, the latter being less well characterized^1,2^.

Macroautophagy, hereafter referred to as autophagy, starts with the formation of a phagophore, which after elongation and cargo sequestration generates a double-membraned autophagosome. The outer autophagosomal membrane ultimately fuses with a lysosome, and the enclosed cargo material is broken down by lysosomal hydrolases^3^. In yeast, autophagosome formation is dependent on Atg8, which becomes activated through its covalent conjugation to phosphatidylethanolamine (PE). In higher eukaryotes, Atg8 has evolved into the LC3/GABARAP family, consisting of LC3A, LC3B, LC3C, GABARAP, GABARAPL1 and GABARAPL2/GATE-16^3^. The function of LC3B has been most studied but it is becoming clear that even though sharing sequence similarity, the LC3/GABARAP proteins play different roles in both autophagy activity and autophagy-independent processes^4^.

Autophagy-related genes (ATGs) and their associated enzymes form the core molecular machinery of autophagosome formation, which was initially described in yeast^5^ and has subsequently been shown to be conserved in other species including humans^6^. A case in point is ATG7, an E1-like enzyme, which forms a central element of two ubiquitin-like conjugation systems required for the lipidation of LC3/GABARAP^3^. In the first system, ATG7 binds and transfers ATG12 to the E2-like enzyme ATG10, which then transfers ATG12 to ATG5. ATG12-ATG5 in complex with ATG16L1 acts as an E3-like enzyme in the final step of LC3/GABARAP-PE conjugation. In the other conjugation system, the C-terminal domain of ATG7 binds LC3/GABARAP prior to the formation of a thioester bond. LC3/GABARAP is subsequently transferred to the E2-like enzyme ATG3 before being conjugated to PE, a process mediated by ATG12-5/ATG16L1^3^. In the growing phagophore, LC3/GABARAP recruits autophagy cargo binding proteins, such as p62 (SQSTM1), which are degraded along with the cargo upon fusion of autophagosomes with lysosomes. In this study, we have identified an isoform of ATG7 that fails to mediate the lipidation of LC3/GABARAP.

## Results

### Two isoforms of *ATG7* are expressed in various tissues

**Figure 1:**
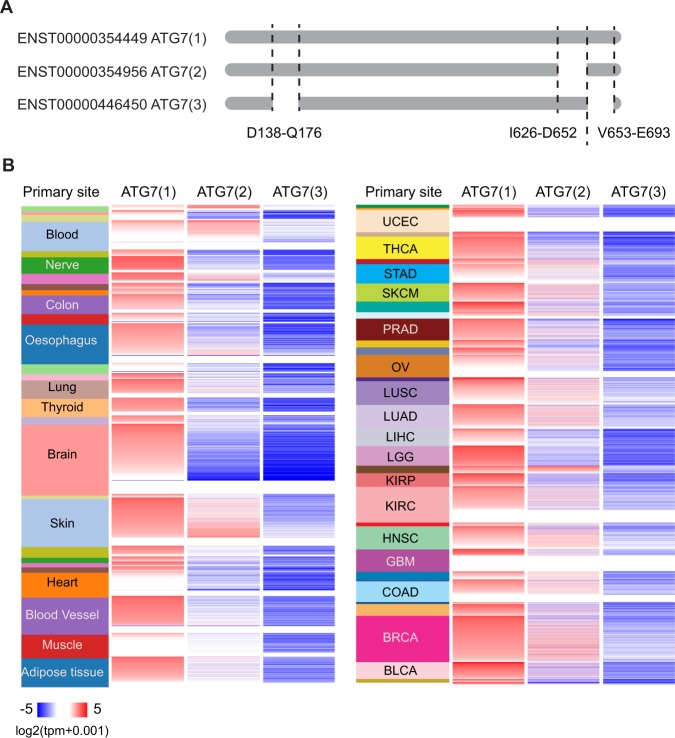
ATG7 isoforms show differential expression pattern. (**A**) Schematic representation of ATG7 isoforms. Location of exons lacking in isoforms 2 and 3 is indicated. (**B**) RNA-seq data from various tissues obtained from GTEX (left panel) and TCGA (right panel) databases were analysed for the expression of ATG7; sample names are indicated on the left, from top to bottom: GTEX: bone marrow, cervix uteri, bladder, fallopian tube, kidney, liver, blood, pituitary, nerve, testis, prostate, small intestine, colon, stomach, oesophagus, pancreas, spleen, lung, thyroid, adrenal gland, brain, salivary gland, skin, breast, vagina, uterus, ovary, heart, blood vessel, muscle, adipose tissue. TCGA: uveal melanoma (UVM), uterine carcinosarcoma (UCS), uterine corpus endometrial carcinoma (UCEC), thymoma (THYM), thyroid carcinoma (THCA), testicular germ cell tumour (TGCT), stomach adenocarcinoma (STAD), skin cutaneous melanoma (SKCM), sarcoma (SARC), rectum adenocarcinoma (READ), prostate adenocarcinoma (PRAD), pheochromocytoma and paraganglioma (PCPG), pancreatic adenocarcinoma (PAAD), ovarian serous cyst adenocarcinoma (OV), mesothelioma (MESO), lung squamous cell carcinoma (LUSC), lung adenocarcinoma (LUAD), liver hepatocellular carcinoma (LIHC), brain lower grade glioma (LGG), acute myeloid leukaemia (AML), kidney renal papillary cell carcinoma (KIRP), kidney clear cell carcinoma (KIRC), head and neck squamous cell carcinoma (HNSC), glioblastoma multiforme (GBM), oesophageal carcinoma (ESCA), diffuse large B-cell lymphoma (DLBC), colon adenocarcinoma (COAD), cholangiocarcinoma (CHOL), cervical and endocervical cancer (CESC), breast invasive cancer (BRCA), bladder urothelial carcinoma (BLCA), adrenocortical cancer (ACC).

According to the Ensembl and RefSeq databases, three protein coding splice variants of human ATG7 exist. Compared to the longest isoform ATG7(1), isoform 2 lacks an exon of 27 amino acids near the C-terminus of the protein, whereas isoform 3 lacks 39 and 41 amino acids from the N- and C- termini, respectively (Fig. 1A, Supplementary Fig. 1A). We analysed the expression of ATG7 isoforms utilizing RNA-sequencing data from the Genotype-Tissue Expression (GTEx) and The Cancer Genome Atlas (TCGA) databases (Fig. 1B). This revealed high expression of ATG7(1), moderate expression of ATG7(2) and low expression of ATG7(3) in the various tissues. Furthermore, we analysed published RNA-sequencing data from human embryonic kidney (HEK293) cells^7^, human hepatocellular carcinoma (HepG2) cells^7^ as well as RNA-sequencing data from normal and tumour samples obtained from human livers^8^. This showed a similar expression pattern, with the highest expression of *ATG7(1)*, lower expression of *ATG7(2)* and lack of *ATG7(3)* (Supplementary Fig. 1B). We next performed qPCR on *ATG7(1)* and *ATG7(2)* in cell lysates obtained from HEK293T cells and the human hepatocellular carcinoma cell lines HepG2 and HuH7, confirming the expression of both isoforms in these cells (Supplementary Fig. 1C).

### ATG7(2) does not bind LC3B

**Figure 2:**
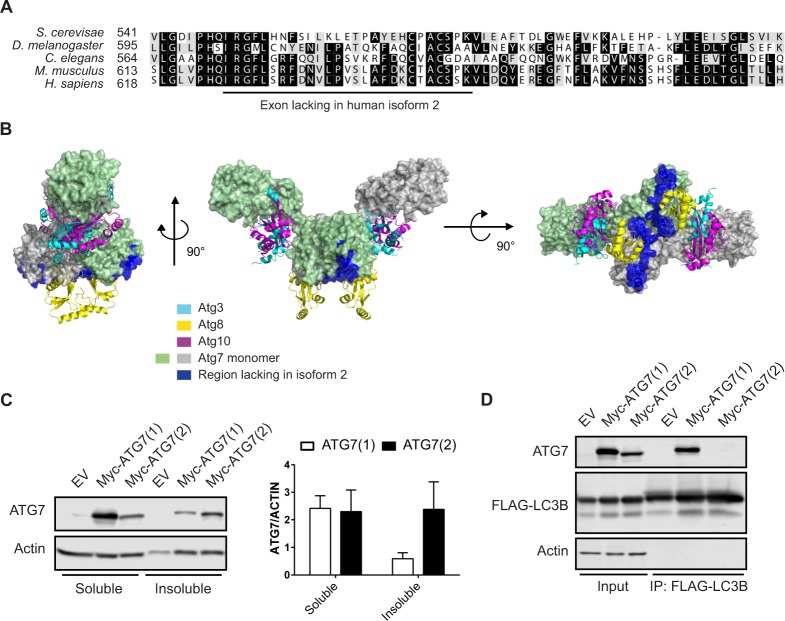
ATG7 isoform 2 is not able to bind LC3. (**A**) Amino acid sequence alignment of ATG7 from *S. cerevisae, D. melanogaster, C. elegans, H. sapiens* and *M. musculus*. The exon lacking in human ATG7(2) is underlined (I626-K652). (**B**) Representation of the yeast Atg7 dimer showing the protomer surfaces colored in faint green or gray. Other ATG proteins, in ribbon representation, are shown to indicate their location in the respective protein complexes. The region lacking in human isoform 2 is shown in dark blue. (**C**) HEK293T cells were transfected with the two isoforms of Myc-tagged ATG7 or empty vector (EV) and protein lysates were prepared using differential detergent extraction prior to Western blot analysis. Membranes were probed with ATG7 and Actin antibodies. Quantification of band intensities was performed using ImageJ software. Error bars represent SEM of four independent experiments. Two-way Anova with Sidak’s multiple comparisons test was performed revealing no significant statistical difference. (**D**) Co-immunoprecipitation experiments in HEK293T overexpressing Myc-tagged empty vector (EV), ATG7(1) or ATG7(2), together with 3xFLAG-LC3. Constructs were detected using ATG7 and FLAG antibodies, respectively.

Alignment of the amino acid sequence lacking in ATG7(2) showed that it is present in species ranging from yeast to human (Fig. 2A). The crystal structure of yeast Atg7 has been resolved in the apo form and in a complex with other Atg proteins, namely Atg3, Atg8 and Atg10^9–13^. The human and yeast ATG7 proteins are highly conserved, sharing approximately 45% sequence identity despite the distant genetic relation between the two species (Supplementary Fig. 2). We thus made use of the yeast Atg7 crystal structure for characterizing a potential role of the exon missing in isoform 2. ATG7 homodimerizes via its C-terminal domain where residues I626-F633 form part of the dimerization interface (Fig. 2B). These amino acids are absent in ATG7(2), suggesting that the homodimerization ability of this short isoform is compromised. In addition, the 27 amino acids that are absent in ATG7(2) (I626-K652) are partially involved in key secondary structure elements, which may affect the overall folding of the domain in which they are encompassed. We therefore hypothesized that the solubility of ATG7(2) might be different from that of ATG7(1). To test this, HEK293T cells were transfected with ATG7(1) or ATG7(2) and protein lysates were prepared using differential detergent extraction^14^. Western blotting revealed that both isoforms were present in the soluble fraction, whereas an increased portion of ATG7(2) was present in the insoluble fraction when compared with ATG7(1) (Fig. 2C, Supplementary Fig. 3A). In yeast, residues R550, F552, H554, L559, L561, T563, P564 and Y566 (which correspond to R627, F629, S631, V636, P638, S640, L641 and F643 in human ATG7) are exposed at the surface and interact directly with Atg8 in the crystal structure of the Atg7/Atg8 complex. These residues are lacking in ATG7(2) and thus the protein would be expected to exhibit decreased binding with the human Atg8 orthologues; LC3/GABARAPs. We tested this by co-transfecting HEK293T cells with FLAG-tagged LC3B and Myc-tagged ATG7(1) or ATG7(2), followed by immunoprecipitation of 3xFLAG-LC3B. Indeed, Western blot analysis revealed that LC3B interacts with ATG7(1) but not with ATG7(2) (Fig. 2D, Supplementary Fig. 3B).

### ATG7(2) is unable to lipidate LC3/GABARAP

**Figure 3:**
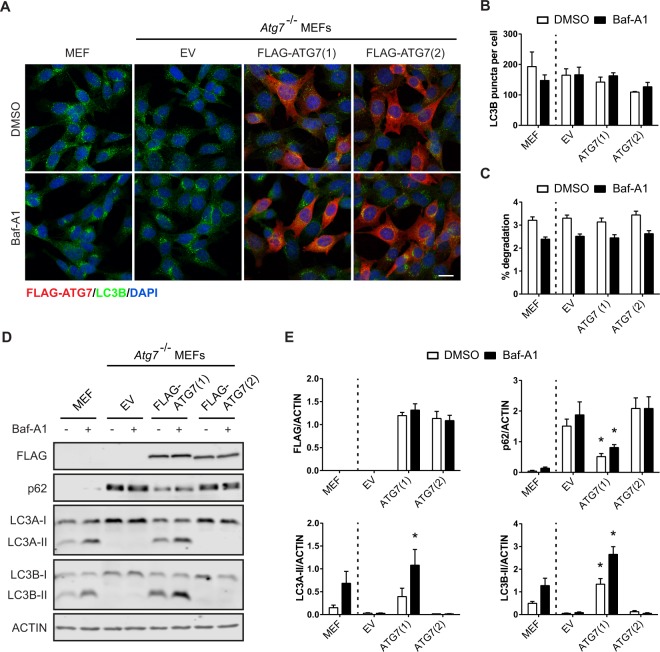
ATG7 isoform 2 fails to lipidate LC3/GABARAP. (**A**) Wild type MEFs or *Atg7*^−/−^ MEFs stably expressing FLAG-tagged empty vector (EV), ATG7(1) or ATG7(2) were treated with Bafilomycin-A1 (Baf-A1) or vehicle control (DMSO) for 4 h and stained with FLAG (red) and LC3B (green) antibodies. Representative images of three experiments are shown. Scale bar represents 20 µm and applies to all images. (**B**) Quantification of LC3B puncta was performed using CellProfiler software. Error bars represent SEM of three independent experiments. Two-way Anova with Sidak’s multiple comparisons test was performed revealing no significant statistical difference. (**C**) Long-lived protein degradation (LLPD) assay of wild type MEFs or *Atg7*^−/−^ MEFs stably expressing FLAG-tagged empty vector (EV), ATG7(1) or ATG7(2), treated with Baf-A1 or DMSO for 4 h. Error bars represent SEM of three independent experiments. Two-way Anova with Sidak’s multiple comparisons test was performed revealing no significant statistical difference. (**D**) Western blot analysis of lysates from wild type MEFs or *Atg7*^−/−^ MEFs stably expressing FLAG-tagged empty vector (EV), ATG7(1) or ATG7(2), treated with Baf-A1 or DMSO for 4 h. Membranes were probed with antibodies against FLAG, p62, LC3A, LC3B and Actin. (**E**) Quantification of band intensities was performed using ImageJ software. Error bars represent SEM of three independent experiments. Two-way Anova with Sidak’s multiple comparisons test was performed to determine statistical significance of ATG7(1) or ATG7(2) expressing *Atg7*^−/−^ MEFs compared to EV *Atg7*^−/−^ MEFs. *p < 0.05.

Given the lost interaction of ATG7(2) with LC3B, we predicted that this isoform would fail to lipidate LC3. To test this, we used *Atg7* knockout mouse embryonic fibroblasts (*Atg7*^−/−^ MEFs) to generate stable cell lines expressing FLAG-tagged human ATG7(1) or ATG7(2). Immunostainings revealed similar expression levels of ATG7(1) and ATG7(2) and both isoforms showed a predominantly cytoplasmic staining pattern (Fig. 3A). We also assessed the expression of endogenous LC3B in the respective cell lines. Lipidated LC3B, which is present at the autophagosomal membrane, is often observed as puncta within the cytoplasm, whereas unlipidated LC3B shows a more diffuse staining^15^. Surprisingly, confocal imaging revealed a similar number of LC3B-positive puncta in both wild type (wt) and *Atg7*^−/−^ MEFs expressing an empty vector (EV) (Fig. 3A,B). Also, no obvious differences in LC3B puncta were observed between the ATG7(1) and ATG7(2) overexpression cell lines. Treatment with the autophagy degradation inhibitor Bafilomycin-A1 (Baf-A1) did not lead to significant changes in the number of LC3B puncta in any of the cell lines when compared to the vehicle control (Fig. 3A,B). Likewise, all cell lines exhibited similar numbers of LC3B puncta under starvation conditions (Supplementary Fig. 4A,B). We also monitored autophagy flux in the presence or absence of Baf-A1 by means of long-lived protein degradation (LLPD) assays. Our data revealed that both wt and *Atg7*^−/−^ MEFs expressing an EV showed comparable levels of protein degradation under basal conditions, which were slightly decreased in the presence of Baf-A1 (Fig. 3C). Overexpression of ATG7(1) or ATG7(2) was not sufficient to further enhance the degradation of long-lived proteins in *Atg7*^−/−^ MEFs.

To assess the lipidation activity of the two ATG7 isoforms, we performed Western blot analyses for members of the LC3/GABARAP family. Of note, the LC3/GABARAP proteins migrate faster in SDS-PAGE gels when lipidated, resulting in two bands with an upper unlipidated (LC3-I) and lower lipidated (LC3-II) protein^15^. As expected, wt MEFs showed two bands each for LC3A (LC3A-I and LC3A-II) and LC3B (LC3B-I and LC3B-II), indicative of functional lipidation (Fig. 3D, Supplementary Fig. 3C). Treatment of these cells with Baf-A1 resulted in an accumulation of the lipidated lower bands of LC3A and LC3B, whereas no lipidation was observed in *Atg7*^−/−^ MEFs, confirming that Atg7 is crucial for the lipidation event. Overexpression of ATG7(1) rescued the lipidation of LC3A and LC3B in *Atg7*^−/−^ MEF cells, whereas the shorter ATG7(2) isoform clearly failed to lipidate the two proteins. A similar pattern was observed for GABARAP (Supplementary Fig. 4C), however, due to low expression and unspecific bands of GABARAP-II, we did not quantify the lipidation of this protein. Finally, we analysed the expression levels of the autophagy cargo receptor p62 in the different cell lines. The *Atg7*^−/−^ MEFs showed increased levels of p62 when compared to the parental cells (Fig. 3D,E, Supplementary Fig. 3C), in line with the inability of the knockout cells to form lipidated LC3. Consistent with that, ATG7(1) expression significantly reduced the levels of p62, whereas ATG7(2) failed to alter p62 expression.

## Discussion

We have identified an isoform of ATG7 that is unable to carry out the best characterized function of the protein, namely the lipidation of LC3/GABARAP. For performing its activity, ATG7 directly interacts with and activates LC3/GABARAP^16^. Sequence alignment and structural analyses revealed that both full-length ATG7(1) and the shorter ATG7(2) isoform contain a critical cysteine residue (C572), which is involved in a thioester bond formation with ATG8 and ATG12, whereas a region important for the initial binding of ATG7 to ATG8 is lost in the short ATG7(2) isoform. Indeed, we did not detect any interactions between ATG7(2) and LC3B. This loss of binding is presumably the reason why ATG7(2) is unable to lipidate LC3/GABARAP. It is also possible that ATG7(2) can bind to other proteins through its C-terminal domain, however, the binding interface has not yet been characterized.

Knockout of either *Atg5* or *Atg7* in mice leads to neonatal death^17,18^, thus highlighting the importance of these genes. Knockdown and overexpression studies of *ATG5* or *ATG7* have revealed various effects on the phenotypes of different cell types, usually ascribed to altered autophagy function. However, an autophagy pathway independent of Atg5 and Atg7 also exists^19^. Mouse embryonic fibroblasts devoid of *Atg5* or *Atg7* are still able to form autophagosomes without lipidated LC3 and carry out some autophagy degradation. Accordingly, wt MEFs and *Atg7*^−/−^ MEFs did not show a difference in the degradation of long-lived proteins at basal conditions. In addition, we did not detect changes in the number of endogenous LC3B puncta when comparing parental and *Atg7*^−/−^ MEFs, or cells overexpressing the two different ATG7 isoforms. However, immunoblotting revealed a clear difference between the four cell lines, with lipidated LC3A and LC3B proteins only appearing in the presence of the full-length ATG7(1) isoform. Together, these findings highlight that caution has to be taken when linking LC3 puncta to the amount of lipidated LC3 or to the number of autophagosomes^20^.

The physiological function of the short ATG7(2) isoform remains unknown and it may serve a role that is unrelated to autophagy. Interestingly, ATG7 has previously been shown to exert functions independent of its lipidation activity, including regulation of the cell cycle and apoptosis through binding of TP53^21^. ATG7(2) might also act as a dominant negative regulator of the full-length ATG7(1) isoform in order to further modulate autophagy activity. Our analysis of GTEx mRNA expression data showed that *ATG7(2)* is highly expressed in blood, which could suggest a role in the hematopoietic system. It is important to note, however, that expression of the different protein isoforms remains to be mapped in both healthy and diseased tissues. Finally, it also requires further investigation how the expression of the different ATG7 isoforms is regulated. In this context it has been shown that abnormal splicing, which leads to a longer *ATG7* transcript, decreases ATG7 protein levels^22^. Importantly, it has to be taken into account that knockdown of *ATG7* mRNA will influence the levels of both *ATG7(1)* and *ATG7(2)*, which can vary between cell types. Further characterizing this shorter ATG7(2) isoform will be important for future autophagy research, both regarding the role of autophagy in healthy cells but also in diseased states.

## Materials and Methods

### Analysis of RNA-seq data

The heatmap for ATG7(1) (ENST00000354449), ATG7(2) (ENST00000354956) and ATG7(3) (ENST00000446450) was generated using Xena Browser^23^. RNA-seq data were selected from Xena data hubs including GTEx (Genotype-Tissue Expression) and TCGA (The Cancer Genome Atlas) data. The samples were sorted based on study type and then by primary sites. For each sample, log2 of the expected count value from RNA-seq data was used for plotting. The results shown here are in part based upon data generated by the TCGA Research Network: http://cancergenome.nih.gov/. The GTEx Project was supported by the Common Fund of the Office of the Director of the National Institutes of Health, and by NCI, NHGRI, NHLBI, NIDA, NIMH, and NINDS. The data used for the analyses described in this manuscript were obtained utilizing the Xena Browser on 08/31/2018.

### Structural analysis of ATG7

The yeast ATG7 apo structure (PDB 3VH2)^9^ was superimposed with yeast complex structures ATG7-ATG8 (PDB 3VH3)^9^, ATG7-ATG3 (PDB 4GSL)^10^ and ATG7-ATG10 (PDB 4GSL)^10^ in Pymol (The PyMOL Molecular Graphics System, Schrödinger, LLC.). Graphics and representation were pursued in Pymol. The following ATG7 protein sequences were obtained from UniProt (http://www.uniprot.org/): *Saccharomyces cerevisiae* P38862, *Caenorhabditis elegans* G5EBK4, *Drosophila melanogaster* Q7JY94, *Mus musculus* Q9D906, *Homo sapiens* isoform 1 O95352, *Homo sapiens* isoform 2 O95352-2, *Homo sapiens* isoform 3 O95352-3. Sequences were aligned using Clustal Omega^24,25^ and BoxShade (http://embnet.vital-it.ch/software/BOX_form.html) was used for converting the alignment file to a PICT file.

### Cell culture and transient transfections

Embryonic fibroblasts from *Atg7* knockout mice (*Atg7*^−/−^ MEFs) were kindly provided by Dr. Masaaki Komatsu and cultured as described previously^18^. HEK293T cells were maintained in DMEM supplemented with 10% FBS. All cell lines were kept at 37 °C in 5% CO_2_ and subcultured to 80% confluency. Transient transfections were performed using FuGENE HD reagent (Promega, Madison, WI) according to the manufacturer’s instructions. The *Atg7*^−/−^ MEF overexpression cell lines were generated by co-transfecting cells with a TRE plasmid containing FLAG-tagged ATG7(1), ATG7(2) or an empty vector (EV), together with plasmids containing the piggybac transposase and reverse tetracycline transactivator rtTA with Neomycin selection. Cells were grown in selection medium with G418 (0.5 µg/µl), which was added to the medium 2 days after transfection. Expression of the constructs was induced by adding 0.1 µg/ml (isoform 1) or 1 µg/ml (EV and isoform 2) doxycycline to the cells for 24 h prior to lysis or fixation.

### Expression constructs

The CMV-ATG7-Myc construct expressing isoform 2 of ATG7 was kindly provided by Dr. Toren Finkel (NIH, Bethesda) and obtained through Addgene (#24921)^26^. The full-length ATG7(1) was generated using the CMV-ATG7(2)-Myc as a template and the Q5 Site-directed Mutagenesis Kit (New England Biolabs, Ipswich, MA) with the following primers: ATG7(1)-F, CAGCCTGGCATTTGACAAATGTACAGCTTGTTCTTCCAAAGTTCTTGATCAATATGAACGAGAAGGATTTAAC and ATG7(1)-R, ACGGGAAGGACATTATCAAACCGTGAAAGAAATCCCCGGATCTGGTGAGGCACAAGCCC. ATG7 was excised from CMV-ATG7-Myc using EcoRI and NotI and cloned into a piggybac vector downstream of a tetracycline response element (TRE). The piggybac constructs were a kind gift from Dr. Kazuhiro Murakami (Hokkaido University)^27^. Constructs were verified by Sanger sequencing. 3xFLAG-LC3B was made using Gateway LR-cloning of p-ENTR-LC3B into pDEST-3xFLAG and generously provided by Trond Lamark (University of Tromso).

### Antibodies

The following antibodies were used for immunofluorescence (IF) and Western blotting (WB): mouse anti-Actin (clone C4, Millipore MAB1501R, WB: 1:10000), rabbit anti-Actin-β (clone 13E5, Cell Signaling Technology 4970, WB: 1:4000), rabbit anti-ATG7 (clone EP1759Y, Millipore 04-1055, WB: 1:10000), mouse anti-FLAG (clone M2, Sigma-Aldrich F3165, IF: 1:5000, WB: 1:5000), rabbit anti-LC3A (clone D50G8, Cell Signaling Technology D50G8, WB: 1:1000), rabbit anti-LC3B (clone D11, Cell Signaling Technology 2775, IF: 1:250, WB: 1:1000), rabbit anti-GABARAP (clone E1J4E, Cell Signaling Technology 13733, WB: 1:1000) and guinea pig anti-p62/SQSTM1 (Progen Biotechnik GmbH GP62-C, WB: 1:1000).

### Immunofluorescence staining and confocal microscopy

Cells were seeded onto 8-well glass slides at a density of 1.5 × 10^4^ cells/well and incubated overnight in the presence of 0.1 µg/ml (isoform 1) or 1 µg/ml (EV and isoform 2) doxycycline. 24 h post-induction, the culture medium was replaced and cells were treated with either 100 nM Bafilomycin (Santa Cruz Biotechnology) or vehicle control (DMSO) for 4 h. Subsequently, cells were fixed with 4% formaldehyde (Thermo Scientific) and incubated with blocking buffer (5% normal goat serum/0.3% Triton X-100 in PBS) for 1 h at room temperature. Following staining with primary antibodies diluted in PBS containing 1% BSA and 0.3% Triton X-100 overnight at 4 °C, cells were incubated with Alexa Fluor 546 or 555 anti-mouse or anti-rabbit IgG secondary antibodies (Life Technologies) for 1 h at room temperature and counterstained with DAPI (Invitrogen, Carlsbad, CA). Finally, slides were mounted in Fluoroshield (Sigma-Aldrich) and images were acquired on a confocal microscope (Olympus FLV1200). Representative images of at least three independent experiments are shown. LC3B puncta were quantified using CellProfiler software and the number of puncta was normalized to the number of nuclei.

### Western blotting

Cells were seeded onto 24-well plates at a density of 5 × 10^4^ cells/well and incubated overnight in the presence of 0.1 µg/ml (isoform 1) or 1 µg/ml (EV and isoform 2) doxycycline. 24 h post-induction, the culture medium was replaced and cells were treated with either 100 nM Bafilomycin (Santa Cruz Biotechnology) or vehicle control (DMSO) for 4 h. Proteins were extracted through whole cell lysis in sodium dodecyl sulphate (SDS) sample buffer (2% SDS, 5% 2-Mercaptoethanol, 10% glycerol, 63 mM Tris-HCl, 0.0025% Bromophenol blue, pH 6.8) and boiling for 5 min at 95 °C. Protein lysates (10–20 µg) were loaded onto 8% or 12.5% gels, separated by SDS polyacrylamide gel electrophoresis (PAGE) and blotted onto methanol-activated polyvinylidene difluoride (PVDF) membranes (Thermo Scientific). After blocking with 5% BSA (Sigma-Aldrich, St. Louis, MO) in Tris-buffered saline containing 0.1% Tween-20, membranes were probed with primary antibodies overnight at 4 °C. DyLight 800 anti-mouse, DyLight 680 anti-rabbit (Cell Signaling Technology) or DyLight 800 anti-guinea pig (LI-COR Biosciences) IgG secondary antibodies were applied for 1 h at room temperature. The blots were scanned with Odyssey imaging system and Image Studio version 2.0 (LI-COR Biosciences). All Western blots were repeated in at least three independent experiments and representative blots are shown for each experiment. Cropped blots are presented in main figures and whole scans are shown in Supplementary Fig. 3.

### FLAG-immunoprecipitation

HEK293T cells were plated onto 5 cm dishes at a density of 1 × 10^6^ cells and incubated overnight, before being co-transfected with Myc-tagged EV, ATG7(1) or ATG7(2) and 3xFLAG-tagged LC3B constructs. The following day (24 h post-transfection), approximately 2 × 10^6^ HEK293T cells per condition were solubilized in lysis buffer (1% Triton-X, 50 mM Tris-HCl pH 7.4, 150 mM NaCl, 1 mM EDTA) supplemented with protease inhibitors (Sigma-Aldrich). The soluble fraction was incubated with ANTI-FLAG M2-Affinity Gel (Sigma-Aldrich) on a roller shaker for 2 h at 4 °C. Proteins bound to the beads were eluted using 3X FLAG peptide at a final concentration of 150 ng/μl (Sigma-Aldrich). After three washing steps, samples were resuspended in 2X SDS sample buffer and subjected to Western blotting.

### Aggregation assay

To monitor the formation of protein aggregates, differential detergent extraction was carried out as previously described^14^. Briefly, soluble proteins were first extracted from transiently transfected HEK293T cells using a mild lysis buffer (1% Triton X-100, 50 mM NaCl, 10 mM Tris pH 7.5, 5 mM EDTA), followed by centrifugation at 16,000 × *g* for 10 min at 4 °C. The pelleted fraction was then washed with PBS and solubilized using a harsher lysis buffer (2% SDS, 50 mM Tris pH 7.5, 1 mM EDTA) and sonication (medium power, 30 sec on/30 sec off) for 5 min using a Bioruptor ultrasonicator (Diagenode). Finally, samples were resuspended in 2X SDS sample buffer and subjected to Western blotting.

### Long-lived protein degradation assay

Cells were grown in 24-well plates for 48 h in RPMI and 10% FBS supplemented with 10 mM ^14^C-L-Valine and appropriate concentrations of doxycycline. Subsequently, cells were washed with PBS and grown in RPMI and 10% FBS supplemented with 10 mM L-Valine for 16 hours. The medium was then changed to a) RPMI, 10 mM Valine and vehicle control (DMSO), b) RPMI, 10 mM Valine and 100 nM Baf-A1, c) HBSS (#14025-050, Gibco), 10 mM Valine and vehicle control (DMSO) or d) HBSS, 10 mM Valine and 100 nM Baf-A1. After 4 hours, the supernatant was collected, 50% TCA added and proteins precipitated overnight at 4 °C. The cells were lysed with 0.2 M KOH overnight at 4 °C. Supernatants were centrifuged and moved into a new tube, the precipitate dissolved and moved to the same sample cell lysate. Both supernatant and lysate were transferred to counting vials and mixed with 3 mL scintillation fluid (Ultima Gold #6013321, Perkin Elmer). ^14^C levels were measured in each sample using a Packard Liquid Scintillation Analyser. The percentage of degradation was determined by comparing the amount of ^14^C in the supernatant to the total ^14^C levels (supernatant and lysate).

### Statistics

All data are expressed as mean ± SEM. Statistical analyses were conducted using GraphPad Prism 6 (GraphPad Software). Analysis of more than two groups was performed using a two-way ANOVA followed by Sidak’s multiple comparisons test. P-values lower than 0.05 were considered statistically significant.

## Electronic supplementary material

Supplementary information
