# Publisher Correction: A short isoform of ATG7 fails to lipidate LC3/GABARAP

**DOI:** 10.1038/s41598-018-35540-y

**Published:** 2018-11-16

**Authors:** M. H. Ogmundsdottir, V. Fock, L. Sooman, V. Pogenberg, R. Dilshat, C. Bindesbøll, H. M. Ogmundsdottir, A. Simonsen, M. Wilmanns, E. Steingrimsson

**Affiliations:** 1 Department of Biochemistry and Molecular Biology, Biomedical Center, Faculty of Medicine, University of Iceland, Sturlugata 8, 101, Reykjavik, Iceland; 2 European Molecular Biology Laboratory, Hamburg Unit, Notkestrasse 85, 22607 Hamburg, Germany; 3 Department of Molecular Medicine, Institute of Basic Medical Sciences, University of Oslo, Sognsvannsveien 9, N-0317 Oslo, Norway; 4 Cancer Research Laboratory, Biomedical Center, Faculty of Medicine, University of Iceland, Sturlugata 8, 101, Reykjavik, Iceland

Correction to: *Scientific Reports* 10.1038/s41598-018-32694-7, published online 26 September 2018

In the original HTML version of this Article, H. M. Ogmundsdottir was incorrectly listed as one of the corresponding authors. The correct corresponding authors for this Article are M. H. Ogmundsdottir and E. Steingrimsson. Correspondence and request for materials should be addressed to mho@hi.is or eirikurs@hi.is. This error has now been corrected in the HTML version of the Article; the PDF version was correct from the time of publication.

