# Supplementary material for: A short isoform of ATG7 fails to lipidate LC3/GABARAP
Source: Sci Rep. 2018 Sep 26;8:14391. doi: 10.1038/s41598-018-32694-7 (PMC6158294; doi:10.1038/s41598-018-32694-7)
Supplement: Supplementary file 1 — Supplementary information [file 41598_2018_32694_MOESM1_ESM.docx]

**A short isoform of ATG7 fails to lipidate LC3/GABARAP**

Ogmundsdottir, M.H.^1*^, Fock, V.^1*^, Sooman, L.^1^, Pogenberg, V.^2^, Dilshat, R.^1^, Bindesboll, C.^3^, Ogmundsdottir, H.M.^4^, Simonsen, A.^3^, Wilmanns, M.^2^, Steingrimsson, E.^1^

Affiliations:

^1^Department of Biochemistry and Molecular Biology, Biomedical Center, Faculty of Medicine, University of Iceland, Sturlugata 8, 101 Reykjavik, Iceland.

^2^European Molecular Biology Laboratory, Hamburg Unit, Notkestrasse 85, 22607 Hamburg, Germany.

^3^Department of Molecular Medicine, Institute of Basic Medical Sciences, University of Oslo, Sognsvannsveien 9, N-0317 Oslo, Norway.

^4^Cancer Research Laboratory, Biomedical Center, Faculty of Medicine, University of Iceland, Sturlugata 8, 101 Reykjavik, Iceland.

^*^ These authors contributed equally to the work.

Corresponding authors: Margret H. Ogmundsdottir, [mho@hi.is](mailto:mho@hi.is), +354-525-5825, and Eirikur Steingrimsson, [eirikurs@hi.is](mailto:eirikurs@hi.is), Tel: +354-525-4270.

**Sup Fig 1**


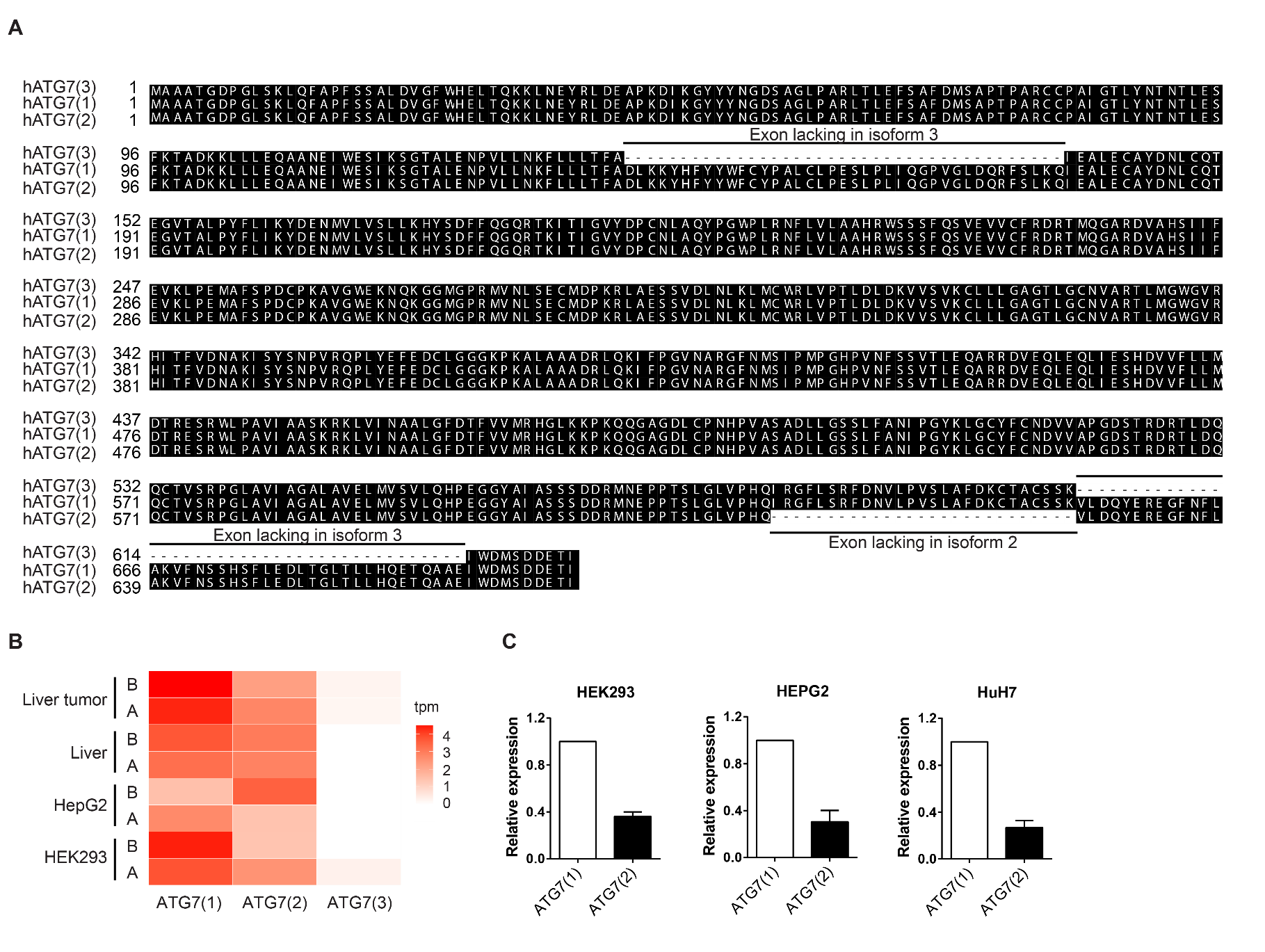


**Sup Fig 2**

**
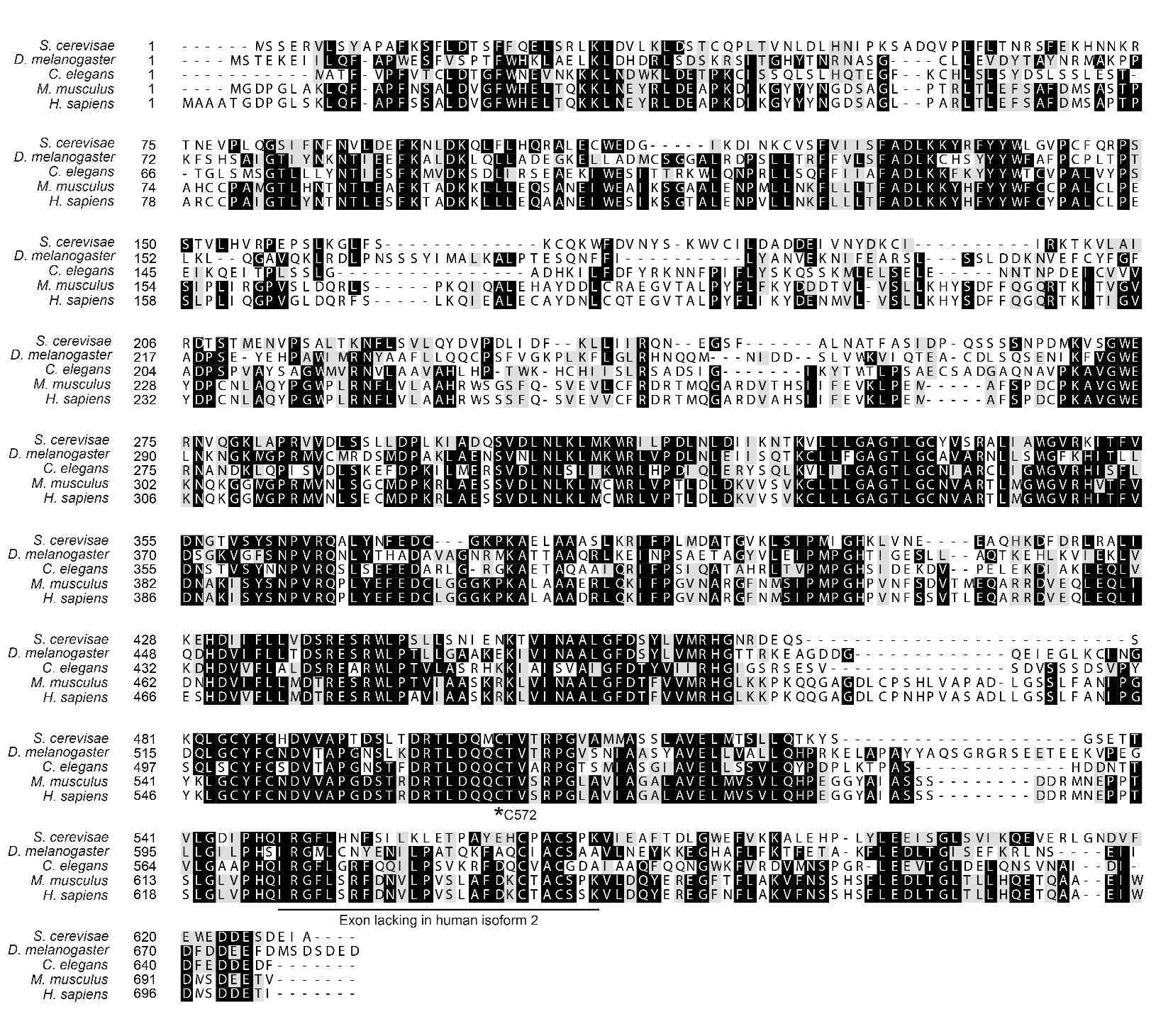
**

**Sup Fig 3**

**
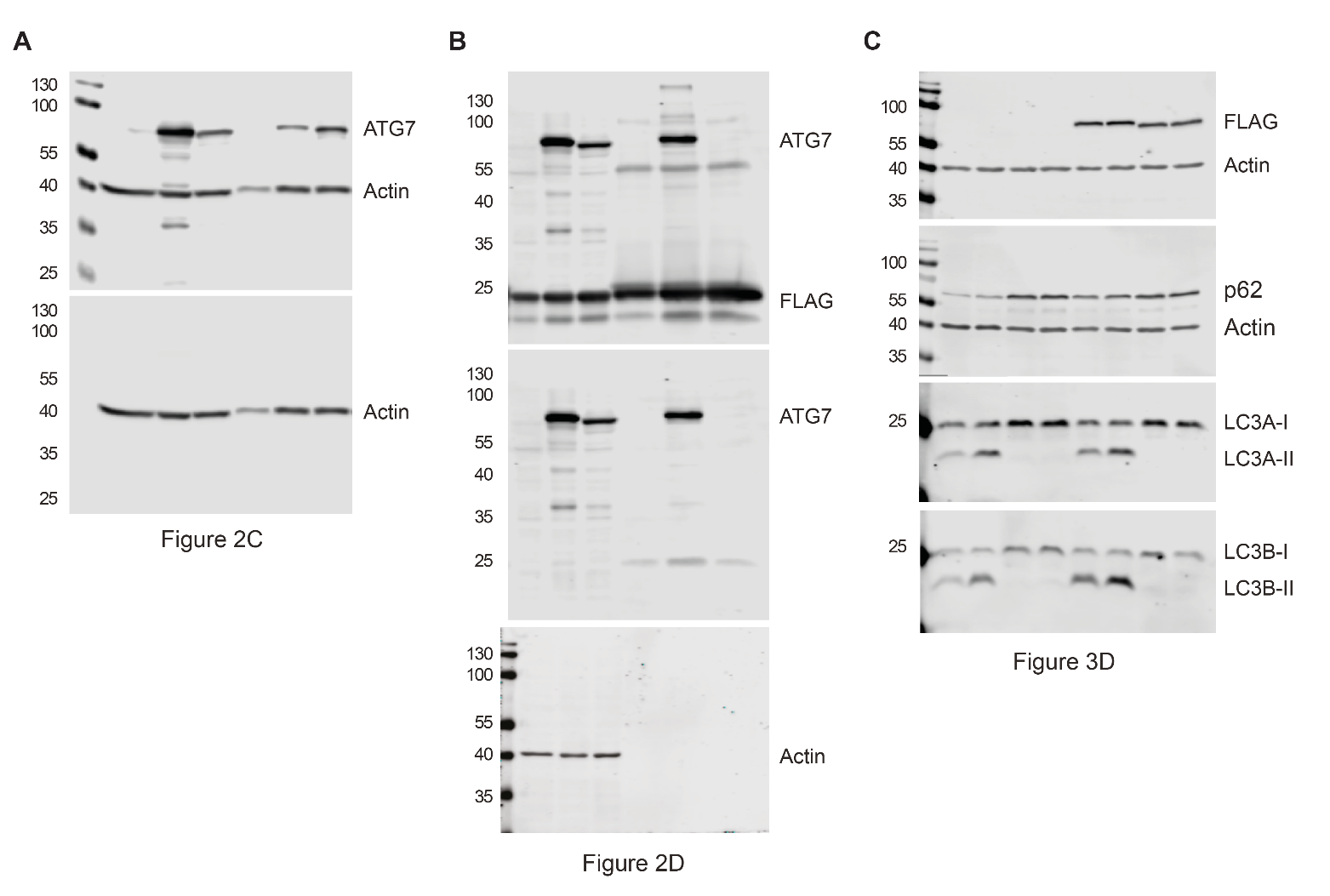
**

**Sup Fig 4**


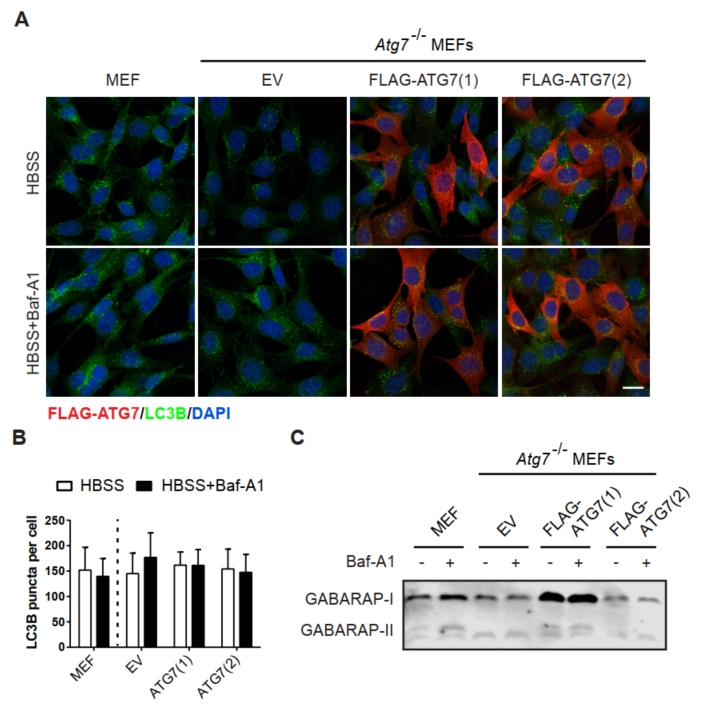


**Supplementary Figure legends**

**Supplementary Figure 1. ATG7 isoforms 1 and 2 are expressed in human liver cells.**

**A)** Sequence alignment of human ATG7 isoforms reveals that compared with full-length ATG7(1), ATG7(2) lacks 27 amino acids towards the C-terminus of the protein and ATG7(3) lacks 39 amino acids towards the N-terminus and 41 amino acids from the C- terminus.

**B)** RNA-seq data from the Protein Atlas databank for two replicates of HEK293 and HepG2 cells, and two replicates of normal and tumour liver samples were analyzed for expression of ATG7. Isoforms indicated are ATG7(1) (ENST00000354449), ATG7(2) (ENST00000354956) and ATG7(3) (ENST00000446450). The raw RNA-Seq data for HEK293 (SRR629569, SRR629570)^1^, HEPG2 (SRR629576, SRR629573)^1^, normal liver tissue (SRR316212, SRR316213)^2^ and liver tumour (SRR316214, SRR316215)^2^ were downloaded from ENA archive (https://www.ebi.ac.uk/ena). Each data set includes two replicates, labelled A and B. RNA-seq reads were mapped to the transcriptome (H.Sapiens.GRCh38, Ensemble) using Kallisto^3^ set at 100 bootstraps and differential expression analysis was performed in R/Bioconductor using the Sleuth package (pachterlab.github.io/sleuth/).

**C)** qPCR data showing expression of ATG7(1) and ATG7(2) in HEK293T, HepG2 and HuH7 cells.

**Supplementary Figure 2. Amino acid alignment of ATG7.**

Alignment of the amino acid sequence of ATG7 in *S. cerevisae*, *D. melanogaster*, *C. elegans*, *M. musculus* and *H. sapiens*. The exon lacking in human isoform 2 is labelled, as well as residue C572 which forms a thioester bond with LC3/GABARAP.

**Supplementary Figure 3. Full scans of Western blots presented in main Figures 2C, 2D and 3D.**

**A)** Western blots presented in Figure 2C. The upper membrane was probed with antibodies against Actin and ATG7, the lower blot shows a single channel image of Actin.

**B)** Western blots presented in Figure 2D. The upper membrane was probed with antibodies against ATG7 and FLAG, the middle blot shows a single channel image of ATG7. The bottom image shows Actin staining.

**C)** Western blots presented in Figure 3D. The upper membranes were probed with antibodies against FLAG and Actin or p62 and Actin, respectively. The lower images represent two membranes probed with antibodies against LC3A or LC3B.

**Supplementary Figure 4. ATG7 isoforms 1 and 2 show a similar expression pattern under starvation conditions.**

**A)** Wild type MEFs or Atg7^-/-^ MEFs stably expressing FLAG-tagged empty vector (EV), ATG7(1) or ATG7(2) were grown in HBSS, treated with Bafilomycin-A1 (Baf-A1) or vehicle control (DMSO) for 4 h and stained with FLAG (red) and LC3B (green) antibodies. Representative images of three experiments are shown. Scale bar represents 20 µm and applies to all images.

**B)** Quantification of LC3B puncta was performed using CellProfiler software. Error bars represent SEM of three independent experiments. Two-way Anova with Sidak’s multiple comparisons test was performed revealing no significant statistical difference.

**C)** Western blot analysis of lysates from wild type MEFs or Atg7^-/-^ MEFs stably expressing FLAG-tagged empty vector (EV), ATG7(1) or ATG7(2), treated with Baf-A1 or DMSO for 4 h. Membranes were probed with an antibody against GABARAP.

References:

1 Uhlen, M. *et al.* Proteomics. Tissue-based map of the human proteome. *Science* **347**, 1260419, doi:10.1126/science.1260419 (2015).

2 Lin, K. T., Shann, Y. J., Chau, G. Y., Hsu, C. N. & Huang, C. Y. Identification of latent biomarkers in hepatocellular carcinoma by ultra-deep whole-transcriptome sequencing. *Oncogene* **33**, 4786-4794, doi:10.1038/onc.2013.424 (2014).

3 Bray, N., Pimentel, H., Melsted, P. & Pachter, L. Near-optimal RNA-Seq quantification. *arXiv:1505.02710*.
